# Thermal cycling of DNA devices via associative strand displacement

**DOI:** 10.1093/nar/gkz844

**Published:** 2019-10-04

**Authors:** Jaeseung Hahn, William M Shih

**Affiliations:** 1 Division of Health Sciences and Technology, MIT, Cambridge, MA 02139, USA; 2 Department of Cancer Biology, Dana Farber Cancer Institute, Boston, MA 02115, USA; 3 Biological Chemistry and Molecular Pharmacology, Harvard Medical School, Boston, MA 02115, USA; 4 Wyss Institute for Biologically Inspired Engineering, Harvard University, Boston, MA 02115, USA

## Abstract

DNA-based devices often operate through a series of toehold-mediated strand-displacement reactions. To achieve cycling, fluidic mixing can be used to introduce ‘recovery’ strands to reset the system. However, such mixing can be cumbersome, non-robust, and wasteful of materials. Here we demonstrate mixing-free thermal cycling of DNA devices that operate through associative strand-displacement cascades. These cascades are favored at low temperatures due to the primacy of a net increase in base pairing, whereas rebinding of ‘recovery’ strands is favored at higher temperatures due to the primacy of a net release of strands. The temperature responses of the devices could be modulated by adjustment of design parameters such as the net increase of base pairs and the concentrations of strands. Degradation of function was not observable even after 500 thermal cycles. We experimentally demonstrated simple digital-logic circuits that evaluate at 35°C and reset after transient heating to 65°C. Thus associative strand displacement enables robust thermal cycling of DNA-based devices in a closed system.

## INTRODUCTION

Toehold-mediated DNA strand displacement (DSD) has enabled construction of sophisticated molecular devices fueled by DNA (1–3). The well-characterized thermodynamics of DNA hybridization (4) allows quantitative design of structures and dynamics of such enzyme-free molecular devices. While expanding the capability and robustness of DSD-based systems (5,6), DNA nanotechnologists have also begun to develop applications of DSD such as label-free single-nucleotide polymorphism genotyping (7), microRNA detection (8) and directed chemical synthesis (9). However, molecular devices fueled by DNA often lack reliable and facile means to refuel for repeated operations, except by automated fluid mixing or exchange, which often is unavailable in a typical lab setting (10). In this work, we present a mechanism that utilizes temperature cycling to reset DNA devices for repeated operation.

Toehold-mediated DSD is a process through which two strands with complementarity hybridize and displace one or more pre-hybridized strands via branch migration catalyzed by toehold binding at the single-stranded region (11). Once equilibrium is reached, cycling back to the original state usually is not possible through external introduction of energy (e.g. via electricity or heat) to the system unless reformation of the initial state is designed to be kinetically favored. Instead, addition of reactant DNA strands can refuel the system for repeated operation (1). However, the resulting accumulation of double-stranded DNA (dsDNA) waste products and further dilution of components leads to deterioration of performance.

To circumvent the drawbacks of adding extra reactant species, DNA devices can be cycled by continuous exchange of buffer to replenish fuel strands and remove waste products (10). Such a system requires immobilization of DNA devices and any memory elements on a surface for the exchange and also requires a fluidic system for automation. In contrast, a reset mechanism that works on a closed system enables operation in bulk solution or else enclosed physical compartments (e.g. vesicles). Alternatively, other mechanisms with orthogonal energy sources have been developed to manipulate such systems more easily. For example, chemical modification of DNA with azobenzene allows the use of photons to direct hybridization and dehybridization (12). However, automated operation of such systems requires chemical modification of DNA strands and high-powered illumination. Alternatively, ions have been used to fuel DNA devices that contain i-motif (13), G-quadruplex (14), or triplex (15) motifs. However, the DNA sequence is constrained for these devices, and a similar fluidic system often is necessary for automated buffer exchange. Thus currently available approaches for repeated operation of DNA devices often require specialized equipment, chemical modification of DNA, and/or stringent constraints on DNA sequence.

Due to the temperature dependence of DNA base pairing, temperature cycling can be used to drive DNA-based systems into hybridized and dehybridized states. For instance, polymerase chain reaction (PCR) amplifies target DNA duplexes through cycles of dehybridization of DNA template duplexes at the melting temperature, hybridization of DNA primers to complementary regions in the DNA template at the annealing temperature, and extension of DNA primers via DNA polymerase (16,17). Heating followed by rapid cooling enables kinetic selection of initial states with less base pairing. For DSD systems, a need to rely on kinetic schemes for resetting to a state with fewer base pairs provides a cumbersome constraint. An alternate method was provided by Rogers and Manoharan, who demonstrated temperature-dependent equilibrium phase transitions of DNA-grafted microparticles programmed by carefully tuning enthalpic and entropic changes (e.g. net base pairing versus net strands released) due to competing DSD reactions (18). The authors accomplish the fine-tuning of these thermodynamic properties via addition of DNA strands to the buffer that compete with grafted strands through DSD. With only two DSD reactions, the authors demonstrated arbitrarily wide gas-solid coexistence, re-entrant melting, and reversible transitions between distinct crystal phases. Utilizing a similar scheme, re-entrant formation of a DNA hydrogel (i.e. solidifies at higher temperatures, disintegrates at lower temperatures) has been demonstrated as well (19).

Here, we have developed temperature-dependent associative DSD (TAD) for reversible strand displacement via thermal cycling. TAD uses cooperative association of multiple strands or strand domains to enable tuning of the enthalpy and entropy changes of complex formation and strand displacement. Thus our design is an adaptation of Rogers and Manoharan's strategy, which focused on multivalent interactions between DNA-grafted microparticles, now in our case to drive DNA devices based on displacement of a single strand from another strand. We explored various design parameters, such as strand concentrations and the net increase of base pairs, in terms of their effect on the system's function. Next, we demonstrated the use of TAD in a simple DNA circuit. TAD does not require any special modification of DNA, does not have sequence constraints beyond orthogonal complementarity based on Watson-Crick base pairing, and does not require specialized equipment except for thermal cyclers, which are widely available in a typical lab setting. Therefore, TAD can be easily implemented for thermal cycling, and potentially can be combined with other external clocking schemes [light (12) or pH (13) responsiveness], to increase the diversity of external control options for DSD-based systems towards programming sophisticated behaviors.

## MATERIALS AND METHODS

All DNA strands were purchased from Integrated DNA Technologies (IDT) with the purification options (SD = standard desalting, HPLC = high performance liquid chromatography) listed in Supplementary Table S1 along with strand name and sequence (see SI1.1). The DNA strands were suspended at around 200–1000 mM in Millipore purified water and stored at −20°C. The stock concentrations were determined by measuring the absorbance of light at a wavelength of 260 nm (*A*_260_) with the extinction coefficient (Ext) for each strand provided by IDT using the Beer-Lambert law ([DNA] = A260/Ext).

We prepared samples in 5 mM Tris/1 mM EDTA buffer (1× TE) with 10 mM MgCl_2_. All samples included incumbent and substrate pair (I–S). I_1_ and S_1_ pair (I_1_–S_1_) conjugated with Cy3 at 5′-end and BHQ2 at 3′-end, respectively, was added at final concentrations of 1 μM for both. I_2_ and S_2_ pair (I_2_–S_2_) conjugated with IABkFQ at 3′-end and Atto488 at 5′-end, respectively, was added at final concentrations of 10 μM for I_2_ and 1 μM for S_2_. Except for the experiment to determine the effect of concentration of AC strands (*C*), TAD strands were added at 10 μM each. To subtract background and normalize the data, we included negative control (I_1_–S_1_ or I_2_–S_2_ only) and positive control (I_1_–S_1_ with 10 μM of complementary strand to S_1_, or I_2_–S_2_ with 10 μM of complementary strand to S_2_) corresponding to 0% and 100% displacement, respectively, for each experiment. Reactions were prepared in black 96-well plates (HSP9666; BioRad) at 20 μL/well volume to ensure rapid temperature change. In a typical experiment, we first added 10 μl of I–S at 2× concentration in 2× TE buffer with 20 mM MgCl_2_ per well. Then, 10 μL of AC strand(s) at 2× concentration in water was added to appropriate well. Finally, fluorescence signal at specific temperature was measured using quantitative PCR (qPCR) machines (BioRad CFX96 C1000 series).

For characterization of TAD, five replicates were prepared for each sample condition to account for random variation in fluorescence signal including the controls. Melting curves were typically obtained by measuring fluorescence signal from 5°C to 65°C at 1°C resolution with specified incubation time before each measurement. If the operation range was at higher temperature, the protocol was adjusted to measure signal at higher temperature range. The same protocol with decreasing temperature was applied before the measurement of melting curve to anneal the sample and also confirm that the fluorescence signal was measured in equilibrium at each temperature point. If the annealing and melting curves showed hysteresis-like behavior, the melting curve was measured using longer incubation time. The obtained data were processed to subtract background, normalize signal, and fit melting curve (see SI1.2 and Supplementary Figure S1). Upon derivation of displacing temperature (*T*_d_) and displacing range (Δ*T*) from the melting curve, the kinetics and repeated operation were measured at the determined temperatures. The same procedure was used to test two orthogonal TAD operations with both I_1_–S_1_ and I_2_–S_2_ in the same solution.

For TAD circuits, we prepared reporter (I_1_–S_1_) strands at 1 μM each, gate and output strands at 2 μM each, all AC strands at 10 μM each, and threshold strand at the specified concentration (0.6 or 1.2 μM) in the final sample solution to be measured. The circuit was reset at 65°C and operated at 35°C. First, the circuit response to varying concentration of input strand was measured by adding 0 to 1 μM of input strand at the 0.1 μM increment in the presence of 0.6 μM of threshold strand. The fluorescence signal was measured every minute. The fluorescence signal from each sample after 8-hour operation at 35°C was used to produce input-output relation plot. The circuit was used to operate OR and AND function by using 0.6 and 1.2 μM of threshold strand, respectively. The qPCR machine was programmed to incubate samples at 65°C for 5 min and 35°C for 8 h for two cycles while measuring fluorescence signal every minute. The input strand was added at 1 and 2 μM to simulate the two-input OR and AND signals, respectively.

## RESULTS

### Design and working principles of TAD

**Figure 1.**
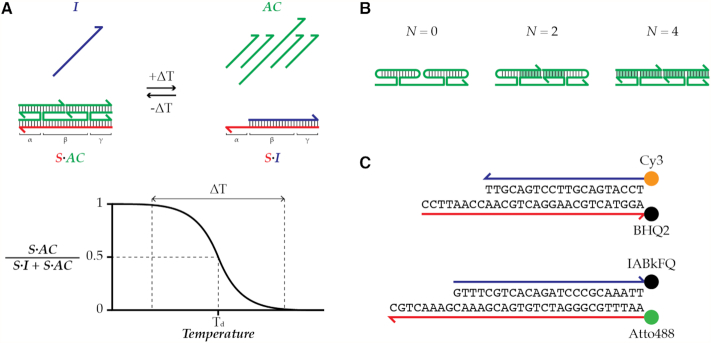
Illustrations showing the principle of TAD and different designs. (**A**) A schematic showing the working principle of TAD. Below temperature T_d_, association of AC strands or strand domains that leads to displacement I-S pair is favored; above temperature T_d_, the reverse is favored. (**B**) Three different AC designs with a pair of four-way junctions. *N* is the number of blunt-end stems (green highlight). (**C**) Two sets of I-S pairs with orthogonal sequences and fluor/quencher pairs were used to verify and quantify TAD operation to enable multiplexed operation in Figure 4.

Our design of TAD was inspired by the principles of remote toehold (20) and associative toehold activation (21,22). At low temperatures, associative complex (AC) strand or strand domains can collectively hybridize to α, β and γ domains of a substrate strand (S) and displace an incumbent strand (I), driven forward by a net increase in base pairing (Figure 1A; also see SI2 and Supplementary Figure S2). The system reverses when the temperature increases to the point where the diminishing energetic impact of the net base pairing no longer can compensate for the loss of entropy from sequestering the multiple AC strands or strand domains on the substrate.

For many applications, it would be desirable for the strand-exchange reaction to exhibit a sharp temperature response. The slope of this response is determined by the enthalpy change, which is set mainly by the net change in base pairing. Therefore, we were motivated to design a displacement reaction driven by a large net increase in base pairing. Towards this end, we conceived TAD as four-way-junction-based complexes that form between S and component AC strands or strand domains in an all-or-nothing fashion (Figure 1A; also see SI3). In this way, net base pairing can be greater than the length of toehold α. To investigate this design space, we prepared a series of TAD systems with varying net base pairing and numbers of strands and characterized their thermal responses in terms of *T*_d_, where 50% of I is displaced from S, and displacing range (Δ*T*), the temperature interval spanning 1–99% displacement of I from S. *T*_d_ represents the balance between enthalpy and entropy, and will be affected by the net number of base pairs, the net number of strands, and the concentrations of those strands. Δ*T* represents the slope of temperature-dependent free energy change dictated by enthalpy change. Larger enthalpy change of the system (e.g. larger net base pairing) therefore results in narrower Δ*T*.

### Characterization of TAD designs

**Figure 2.**
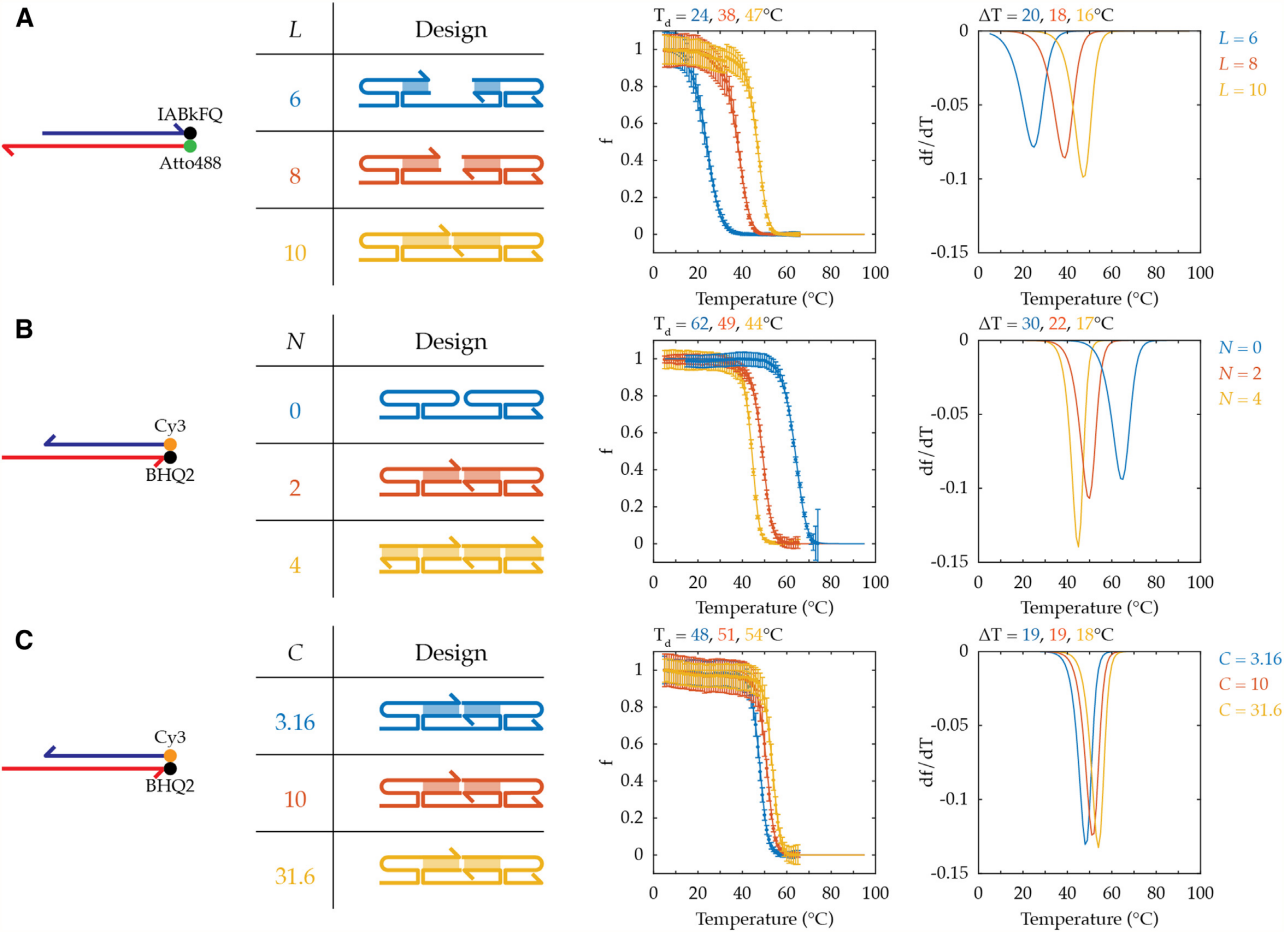
Characterization of TAD. Fluorescence signal is measured as a surrogate for fraction of I-S pair displaced to determine the effect on *T*_d_, temperature at 50% displacement, and ΔT, temperature interval for 1–99% displacement, of varying design parameters: (**A**) *L*, length of blunt-end stems fixing the number of stems at two (*L* = 6, 8, or 10; *N* = 2, *H_L_* = 6, *H_l_* = 4, *C* = 10 μM), (**B**) *N*, number of blunt-end stems fixing stem-length at 8 bp (*N* = 0, 2, or 4; *L* = 8, *H_L_* = 6, *H_l_* = 4, *C* = 10 μM). (**C**) *C*, concentration in μM of each AC strand fixing *L* = 8 bp and *N* = 2 stems (*C* = 3.16, 10, or 31.6 μM; *N* = 2, *L* = 8, *H_L_* = 6, *H_l_* = 4). The experimental data (dot) of displacement fraction, f, were fitted to obtain the melting curve (line), and the first derivative with respect to temperature, d*f*/d*T*, was plotted to visualize the sharpness of transition. Error bars represent standard deviation.

By utilizing I and S conjugated with quencher and fluorophore, the activity of TAD was measured from the fluorescence signal, which is directly proportional to displacement of quencher and fluorophore strands. To verify the wide applicability of TAD, we used two sets of I-S pairs with different lengths (see SI4 for choice of designs shown in Figure 2). First we tested three designs that each have two blunt-end stems and differ only by the length of those stems (*L*), which changes the net base pairing for displacement (Figure 2A, left). For these designs with *N* = 2, two hairpin structures were required to form four-way junctions between α, β, and γ domains (Figure 2A, left). Unless specified, the number of base pair of each hairpin stem (*H*_L_) was 6, and the number of nucleotide of each hairpin loop was 4 in all the AC designs with hairpin structures. As expected, longer blunt-end stem lengths exhibited increased *T*_d_ and decreased Δ*T* (Figure 2A, right). Next we compared three designs that maintain blunt-end stem length at 8 bp but vary the number of those stems (*N*) from 0 to 4 (Figure 2B, left). Here increasing *N* leads to a larger number of non-hairpin (i.e. blunt-end) base pairs in the upper helix, therefore greater enthalpy benefit from displacement. This is so because hairpin (i.e. non-blunt-end) stems can remain base-paired even in the absence of S. Larger *N* also implies a larger number of AC strands, therefore a greater entropy penalty for displacement. Consistent with these expectations, we observed larger *N* decreased both *T*_d_ and Δ*T* (Figure 2B, right; also see SI5 and Supplementary Figures S3 and S4). Conversely, it was surprising to us to find that an AC design with *N* = 0 could be outcompeted by I with the observed *T*_d_ (62°C with *H*_L_ = 6 and *H*_l_ = 4) since the AC-S complex is predicted to make more base pairs than the I–S pair. Unafold (23) predicts stable hairpin structures at this *T*_d_, and the number of strands exchanged is the same (i.e. 1). AC designs with *N* = 0 and hairpin structures with longer stems, and therefore increased stability, were still outcompeted by I with the similar *T*_d_ (see SI6 and Supplementary Figure S5). *T*_d_ decreased only when the hairpin stem was shorter. A possible explanation is that four-way junctions are unstable at higher temperatures (24). In this scenario, the four-way junction penalty is small at low temperature, and the increase in net base pairing mainly drives formation of AC–S complex. At high temperature, the four-way junction penalty outweighs toehold hybridization, and the I-S complex reforms. In addition, increasing *C* of the same design resulted in higher *T*_d_ with similar ΔT (Figure 2C). Therefore by modulating design parameters such as *N, L* and *C*, it is possible to fine-tune TAD properties, *T*_d_ and Δ*T*, for the operation of DNA devices (see Supplementary Table S2 for *T*_d_ and Δ*T* from AC designs tested in this study).

### Repeated operation of TAD

**Figure 3.**
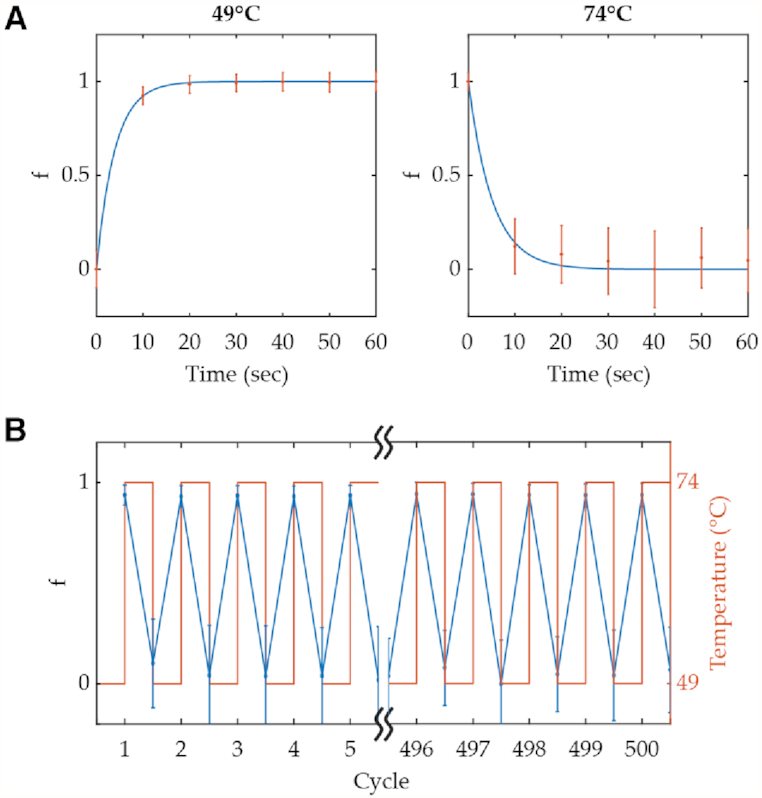
Kinetics and repeated operation of TAD. (**A**) Experimental data (dot) and fitted curve (line) of displacement fraction, f, showing activation (left) and deactivation (right) kinetics of AC design with *N* = 0, *H_L_* = 6, *H_l_* = 4, and *C* = 10 μM for I–S pair conjugated with Cy3-BHQ2 pair. (**B**) Repeated operation of the same sample from panel (A) showing first and last five cycles of a 500-cycle experiment. Error bars here represent standard deviation.

The repeated operation of TAD may be expected to proceed with negligible loss of activity over a large number of cycles because there is no accumulation of waste product or dilution of the sample associated with other mechanisms that require exchange of mass (1) (although after a much greater number of cycles, activity eventually should deteriorate due to accumulation of heat-accelerated DNA depurination damage). To test this hypothesis, first the kinetics of TAD activity of an AC design (*N* = 0; Cy3-BHQ2 pair) at ON (49°C) and OFF (74°C) temperatures corresponding to 99 and 1% displacement, respectively, were measured to determine the cycling time (Figure 3A). Both reactions took less than 30 seconds for this particular design. Nearly identical TAD activity was observed when cycling between ON and OFF temperatures while measuring fluorescence for 500 cycles (Figure 3B).

### Operation of two orthogonal TADs

**Figure 4.**
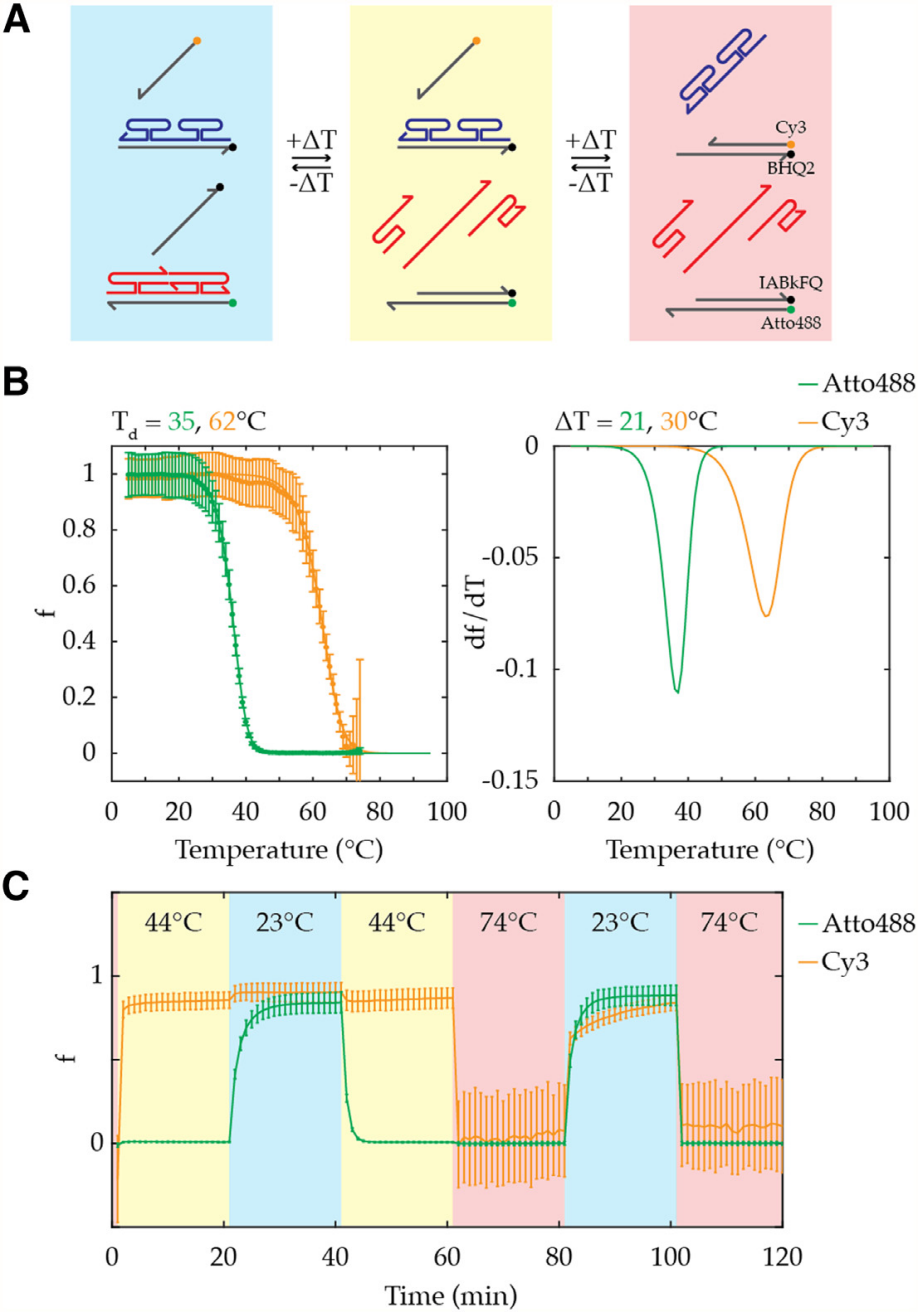
Operation of orthogonal TAD designs in the same solution. (**A**) Programmed states of TAD designs at low (blue), medium (yellow), and high (red) temperature. (**B**) Melting curve and its first derivative verifying the orthogonal operation of two TAD designs (top: *N* = 0, *H_L_* = 6, *H_l_* = 4, and *C* = 10 μM; bottom: *N* = 2, *L* = 8, *H_L_* = 6, *H_l_* = 4, and *C* = 10 μM) in the same solution and showing three accessible states in the system. The experimental data (dot) of displacement fraction, f, are fitted to derive melting curve (line), and its first derivative with respect to temperature, d*f*/d*T*, is plotted to visualize the sharpness of transition. (**C**) Time course trace of f depending on the temperature. Error bars here represent standard deviation.

In principle, multiple TAD operations could be performed in the same reaction by careful sequence design of DNA. As a proof-of-principle, two sets of TAD strands with different *T*_d_ were chosen so that there could be three distinct states in the system: both fluorophores off, one fluorophore on, and both fluorophores on (Figure 4A). Measurement of two-color fluorescence versus temperature (Figure 4B) confirmed TAD activity on two sets of I–S pairs with orthogonal sequences and fluor/quencher pairs and enabled characterization of the corresponding temperature windows for each of the three states (Figure 4B). Programmed temperature jumps could be used to induce switching between states (Figure 4C). Unsurprisingly, TAD operation at lower temperature [Figure 4C, Cy3 design (orange), comparing kinetics from 74°C to 23°C at minutes 80–100 (i.e. lower temperature) versus 74°C to 44°C at minutes 0–20 (i.e. higher temperature)] resulted in slower kinetics.

### TAD-based circuit

**Figure 5.**
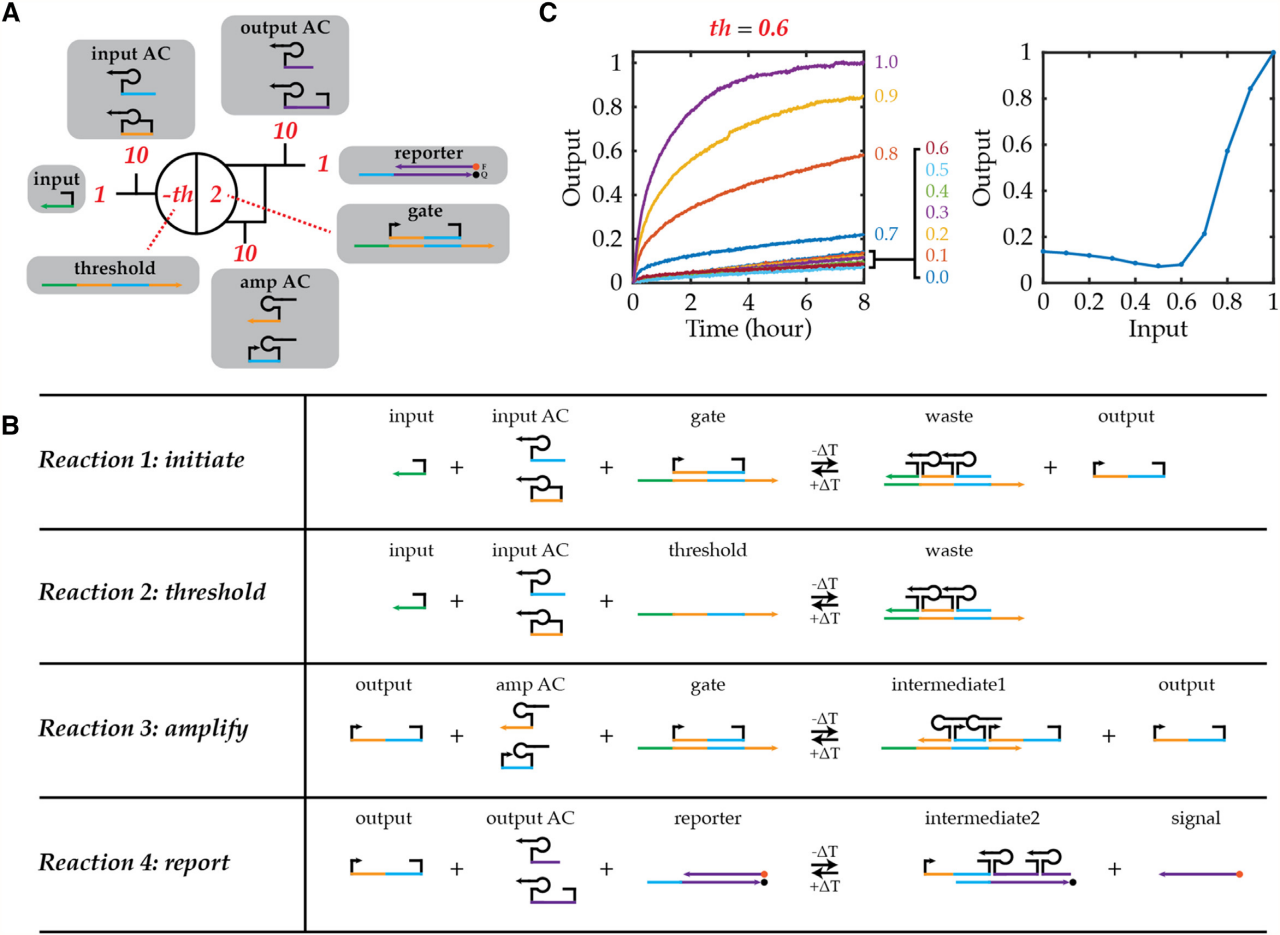
Design and implementation of TAD gate. (**A**) Abstract schematic of the gate. Red numbers indicate relative concentrations of initial DNA species shown in gray boxes. Colored lines indicate DNA domains with distinct sequences for each color; hairpin and blunt-end stem domains have unique sequences but here are all rendered in black. (**B**) Four basic reactions involved in TAD gate: initiation, thresholding, amplification, and reporting. (**C**) Kinetics experiment of TAD gate at varying input-strand concentrations (left). Input-versus-output relationship is determined by plotting output at 8 h against initial input-strand concentration.

The reversible nature of TAD allows refueling of the system via transient temperature jump to build DNA circuits and devices for repeated operations. In the future, such reusable circuits and devices could be programmed to compute and operate differently each cycle with changing input signals [e.g. light-responsive (12) or pH-responsive (13) elements] or memory from past cycles that survives high temperatures (e.g. enzymatic ligation triggered at the end of a cascade). As a proof-of-principle to demonstrate only the refueling behavior (i.e. identical behavior each cycle), a reusable circuit inspired by the DNA seesaw circuit (6) was designed to be compatible with the TAD mechanism and powered by temperature as universal fuel (Figure 5A; also see SI7). This TAD-based circuit does not ‘seesaw’ like the DNA seesaw gate that exchanges activity of DNA signals through the toehold exchange-based reversible reaction. Instead, a TAD reaction acts as an initiation step in the presence of input strand, and another TAD reaction acts as a catalytic cycle that allows signal amplification much like the DNA seesaw circuit (Figure 5B). The circuit was designed to preserve the thresholding capability of DNA seesaw circuits by adding a small excess of gate bottom strands (threshold) over gate top strands (output) to quench the specified amount of input strand. Therefore, the TAD circuit can be reconfigured to perform an OR logic function as well as an AND logic function through concentration adjustment for thresholding that allow universal Boolean function evaluation using dual-rail logic (6). The reporter gate is used to transform the output into a fluorescence signal through another TAD reaction. All reactions are based on TAD, therefore the circuit can be reset via a transient temperature spike.

**Figure 6.**
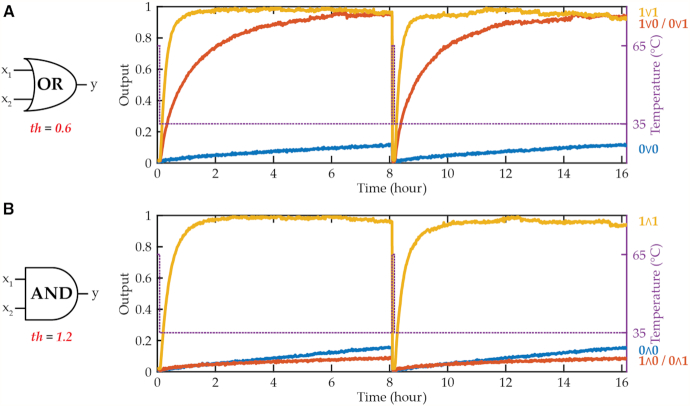
TAD logic gates. Kinetic experiments showing TAD gate operating two-input (**A**) OR and (**B**) AND computation depending on the initial thresholding strand concentration.

The resulting DNA circuit demonstrated input concentration-dependent fluorescence kinetics, and the input-versus-output relationship showed the performance of thresholding (Figure 5C). With the input concentration at or below the threshold concentration, the fluorescence signal was suppressed at or below the circuit leak. Above the threshold concentration, the additional input increased fluorescence kinetics. Adjusting the threshold concentration enabled the OR and AND operations, and renewed operation was possible by simply resetting the circuit at 65°C and re-operating at 35°C (Figure 6). 0.6× and 1.2× concentration of threshold was used for OR and AND gate, respectively, and 1× and 2× concentration of input strand was added to the circuit to simulate the two-input OR and AND signals, respectively. In future implementations, if such a gate were designed to receive two or more input strands coming from other upstream reactions, the input signals would include the input sequence used in this experiment appended to different sequences. The fluorescence kinetics demonstrated OR and AND computations with the same set of molecules varying only the concentrations of input and threshold. In general, the bigger difference between input and threshold concentrations resulted in faster kinetics due to the linear nature of amplification step (i.e. faster amplification in the presence of higher concentration of input).

## DISCUSSION

We have developed a mechanism to reset DSD using temperature cycling, characterized its engineering parameters, and successfully demonstrated the operation of circuit using this mechanism. Importantly, TAD provides a facile means to design, produce, and operate DNA devices that require repeated and extended operation because it can be actuated using readily available thermal cyclers without any special modification of DNA molecules. For future implementations, each cycle could be programmed to have the potential to be distinct, either through additional external inputs [e.g. light irradiation (12) or pH change (13) as input], or else through memory of events in past cycles that persists (e.g. ligation or polymerization at the end of a cycle). Through use in combination with orthogonal actuation mechanisms such as photon- and proton-mediated DSD, TAD has the potential to expand our ability to control DNA-based systems.

In order to use DNA devices that operate robustly through a large number of cycles, a reliable energy source for actuation is required. The first generation of TAD described here represents progress towards this direction, however further improvement to fine-tune TAD properties with faster kinetics, especially at lower temperature, would be helpful (see SI8). Furthermore, the quantitative design of TAD strands would require an advanced understanding of the thermodynamic properties of DNA junctions that we currently lack. Nonetheless, we anticipate that even early implementations of temperature-driven actuation via TAD will prove widely applicable to develop DNA devices with extended lifetime and operation.

## Supplementary Material

gkz844_Supplemental_File

## Notes

Present address: Jaeseung Hahn, Department of Biomedical Engineering, Columbia University, New York, NY 10027, USA.
