# Supplementary material for: Thermal cycling of DNA devices via associative strand displacement
Source: Nucleic Acids Res. 2019 Oct 4;47(20):10968–75. doi: 10.1093/nar/gkz844 (PMC6847259; doi:10.1093/nar/gkz844)
Supplement: gkz844_Supplemental_File [file gkz844_supplemental_file.pdf]

SUPPORTING INFORMATION

Thermal Cycling of DNA Devices via Associative Strand Displacement

Jaeseung Hahn[1,2,3,4], William M. Shih[2,3,4,*]

[1] Division of Health Sciences and Technology, MIT, Cambridge, MA 02139, USA
[2] Department of Cancer Biology, Dana Farber Cancer Institute, Boston, MA, 02115, USA
[3] Biological Chemistry and Molecular Pharmacology, Harvard Medical School, Boston, MA, 02115, USA
[4] Wyss Institute for Biologically Inspired Engineering, Harvard University, Boston, MA, 02115, USA

* To whom correspondence should be addressed. Tel: +1 617 632 5143; Fax: +1 617 632 4393; Email: william_shih@dfci.harvard.edu

Present Address: Jaeseung Hahn, Department of Biomedical Engineering, Columbia University, New York, NY 10027, USA

SI1. Sequences and Data Processing

SI1.1: Sequences

The sequences were designed and analyzed using NUPACK (1). Sequences of all the strands used in this study are listed in SI Table 1. Associative complex (AC) strands nomenclature is as the following:

# = target fluorophore-quencher pair (1 = Cy3-Black Hole quencher 2 (BHQ2); 2 = Atto488-Iowa Black FQ (IABkFQ))

N = number of blunt-end stem in the design (0, 2, or 4)

L = number of base pair of each blunt-end stem in the design (6, 8, or 10)

H_L = number of base pair of each hairpin stem in the design (4, 6, 8, or 10)

H_l = number of nucleotide of each hairpin loop in the design (4 or 10)

SI Table 1. Sequence Data

**Strand**	**Sequence**	**IDT Purification**
Substrate$_1$ (S_1)	CCTTAACCAACGTCAGGAACGTCATGGA/3BHQ_2/	HPLC
Incumbent$_1$ (I_1)	/5Cy3/TCCATGACGTTCCTGACGTT	HPLC
S_1 Full Complement ($S_{1,FC}$)	TCCATGACGTTCCTGACGTTGGTTAAGGTCAACATCGTCTC	SD
Two-strand system anchor	TCCATGACGTTCCT	SD
Two-strand system initiator	GACGTTGGTTAAGG	SD
AC (# = 1, *N* = 0, H_L = 4, H_l = 4) strand	TCCATGACCGAGTATACTCGCGCAAAGTTGCGGTTCCTGACGTTCCACAGTAGTGGCGCTAGATAGCGGGTTAAGG	SD
AC (# = 1, *N* = 0, H_L = 6, H_l = 4) strand	TCCATGACCGCACGTTTACGTGCGCGTCGCCCTCGCGACGGTTCCTGACGTTCGCACCAGATGGTGCGCGTGGCTGTAGCCACGGGTTAAGG	SD
AC (# = 1, *N* = 0, H_L = 8, H_l = 4) strand	TCCATGACCGCTGGACGTGCGTCCAGCGCGTGCGTGGTAACACGCACGGTTCCTGACGTTCGCTGGGAGCAATCCCAGCGGCACTCCGGAGCCGGAGTGCGGTTAAGG	SD
AC (# = 1, *N* = 0, H_L = 10, H_l = 4) strand	TCCATGACCGTCGAGGGCTCTCGCCCTCGACGCGGAGCTGCGCTGTCGCAGCTCCGGTTCCTGACGTTCGCACCTCGGCCAGCCGAGGTGCGGCTGCCTCGGGATACCGAGGCAGCGGTTAAGG	SD
AC (# = 1, *N* = 0, H_L = 4, H_l = 10) strand	TCCATGACCGAGTAAACCTGATCTCGCGCAACTCCTACTATGCGGTTCCTGACGTTCCACTTATTCATCTGTGGCGCTCCAATTCAATAGCGGGTTAAGG	SD
AC (# = 1, *N* = 2, *L* = 6, H_L = 6, H_l = 4) strand 1	GATGGGCGTGGCGAGTGCCACGGGTTAAGG	SD
AC (# = 1, *N* = 2, *L* = 6, H_L = 6, H_l = 4) strand 2	GCACCTGTTCCTGACGTTCCCATC	SD
AC (# = 1, *N* = 2, *L* = 6, H_L = 6, H_l = 4) strand 3	TCCATGACCGTCGCACAAGCGACGAGGTGC	SD

AC (# = 1, N = 2, L = 8, H_L = 6, H_I = 4) strand 1	GGGATTGGCGTGCTCTTGAGCACGGGTTAAGG	SD
AC (# = 1, N = 2, L = 8, H_L = 6, H_I = 4) strand 2	AGTGCTTAGTTCCTGACGTTCCAATCCC	SD
AC (# = 1, N = 2, L = 8, H_L = 6, H_I = 4) strand 3	TCCATGACCGTGCGGATACGCACGTAAGCACT	SD
AC (# = 1, N = 2, L = 10, H_L = 6, H_I = 4) strand 1	AGGTATTGGTAGCCTGGATACAGGCTGGTTAAGG	SD
AC (# = 1, N = 2, L = 10, H_L = 6, H_I = 4) strand 2	CCGAGATCTTGTTCCTGACGTTACCAATACCT	SD
AC (# = 1, N = 2, L = 10, H_L = 6, H_I = 4) strand 3	TCCATGACCGACGCGAGTGCGTCGAAGATCTCGG	SD
AC (# = 1, N = 4, L = 8) strand 1	GTTAGGCAGGTTAAGG	SD
AC (# = 1, N = 4, L = 8) strand 2	CGAAATGGTGCCTAAC	SD
AC (# = 1, N = 4, L = 8) strand 3	CTTGTTCGGTTCCTGACGTTCCATTTCG	SD
AC (# = 1, N = 4, L = 8) strand 4	GCTACTCTCGAACAAG	SD
AC (# = 1, N = 4, L = 8) strand 5	TCCATGACAGAGTAGC	SD
AC (# = 1, N = 6, L = 10, H_L = 6, H_I = 4) strand 1	CGTGCTACTAGCGAGCCCTAGCTCGCGGTTAAGG	SD
AC (# = 1, N = 6, L = 10, H_L = 6, H_I = 4) strand 2	AGATTCAAGGTAGTAGCACG	SD
AC (# = 1, N = 6, L = 10, H_L = 6, H_I = 4) strand 3	GTTGTATGGTCCTTGAATCT	SD
AC (# = 1, N = 6, L = 10, H_L = 6, H_I = 4) strand 4	CGATTTGACTGTTCCTGACGTTACCATACAAC	SD
AC (# = 1, N = 6, L = 10, H_L = 6, H_I = 4) strand 5	CTCATCTCCAAGTCAAATCG	SD
AC (# = 1, N = 6, L = 10, H_L = 6, H_I = 4) strand 6	CGTGAAGTAGTGGAGATGAG	SD
AC (# = 1, N = 6, L = 10, H_L = 6, H_I = 4) strand 7	TCCATGACCCGGTGGATACACCGGCTACTTCACG	SD
Substrate$_2$ (S_2)	/5ATTO488N/AATTTGCGGGATCTGTGACGAAACGAAACTGC	HPLC
Incumbent$_2$ (I_2)	GTTTCGTCACAGATCCCGCAAATT/3IABkFQ/	HPLC
S_2 Full Complement ($S_{2,FC}$)	GCAGTTTCGTTTCGTCACAGATCCCGCAAATT	SD
AC (# = 2, N = 0, H_L = 6, H_I = 4) strand	GCAGTTTCCGTGCCAGTTGGCACGCGAGCGCGATCGCTCGGTTTCGTCACAGATCCGCACCGAACTCGGTGCCGTGCGGTAACGCACGCGCAAATT	SD
AC (# = 2, N = 2, L = 6, H_L = 6, H_I = 4) strand 1	GCAGTTTCCGCAAGGACTCTTGCGTGGGAG	SD
AC (# = 2, N = 2, L = 6, H_L = 6, H_I = 4) strand 2	CTCCCAGTTTCGTCACAGATCCAGTCCA	SD
AC (# = 2, N = 2, L = 6, H_L = 6, H_I = 4) strand 3	TGGACTAGGCAGCCTACTGCCTCGCAAATT	SD
AC (# = 2, N = 2, L = 8, H_L = 6, H_I = 4) strand 1	GCAGTTTCCGGTCACTTGTGACCGTATGGACG	SD
AC (# = 2, N = 2, L = 8, H_L = 6, H_I = 4) strand 2	CGTCCATAGTTTCGTCACAGATCCACCTCCAT	SD

AC (# = 2, N = 2, L = 8, H_L = 6, H_I = 4) strand 3	ATGGAGGTCGTGGCTGCTGCCACGCGCAAATT	SD
AC (# = 2, N = 2, L = 10, H_L = 6, H_I = 4) strand 1	GCAGTTTCCGCACGGAAGCGTGCGTGGCCTAAAG	SD
AC (# = 2, N = 2, L = 10, H_L = 6, H_I = 4) strand 2	CTTTAGGCCAGTTTCGTCACAGATCCACCTCACTTA	SD
AC (# = 2, N = 2, L = 10, H_L = 6, H_I = 4) strand 3	TAAGTGAGGTCGTGCCGTCTGGCACGCGCAAATT	SD
AC (# = 2, N = 4, L = 8) strand 1	GCAGTTTCACAAGGAC	SD
AC (# = 2, N = 4, L = 8) strand 2	GTCCTTGTTATGCTCA	SD
AC (# = 2, N = 4, L = 8) strand 3	TGAGCATAGTTTCGTCACAGATCCATTTCACC	SD
AC (# = 2, N = 4, L = 8) strand 4	GGTGAAATAGTAGAGG	SD
AC (# = 2, N = 4, L = 8) strand 5	CCTCTACTCGCAAATT	SD
AC (# = 2, N = 4, L = 10) strand 1	GCAGTTTCGAGTCTTAGC	SD
AC (# = 2, N = 4, L = 10) strand 2	GCTAAGACTCTAATGACAAC	SD
AC (# = 2, N = 4, L = 10) strand 3	GTTGTCATTAGTTTCGTCACAGATCCACAGCTTAAC	SD
AC (# = 2, N = 4, L = 10) strand 4	GTTAAGCTGTATGAGTAAGG	SD
AC (# = 2, N = 4, L = 10) strand 5	CCTTACTCATCGCAAATT	SD
input	TTCAGGCTTTAGGGACGG	SD
input AC strand 1	TGTGGACTGGTGCGATGACCGACCTTATGGTCGGAGCCTGAA	SD
input AC strand 2	TTGGTTAACGACGCGCAAGCGTCGAGTCCACA	SD
output	AGGATGGATTGGTTAAGGTGCGATGATTCAGAGTGG	SD
amp AC strand 1	CCACTCTGAGACCGCGTACGGTCTGGTTAAGGTGATTCAGGC	SD
amp AC strand 2	GCCTGAATGCAGCGTAGTCGCTGCCGATGATT	SD
output AC strand 1	TGTAGCTTGTTCCTGACGGGTCGGTTCGCCGACCTCCATCCT	SD
output AC strand 2	TCCATGACAAGTTGATGCCAACTTAAGCTACA	SD
threshold	CCGTCCCTAATCATCGCACCTTAACCAATCATCGCA	SD

SI1.2: Data processing

All the data processing was performed using MATLAB. The fluorescence signals from both Cy3 and Atto488 were dependent on temperature, which necessitated the background subtraction and normalization of signal to obtain melting curves (Figure S1). The signals from all the samples were subtracted by the signal from negative control to account for the background signal. Then, the data were normalized by dividing the value with positive control. For the samples with replicates (i.e. TAD characterization samples), we used an average value of five replicates for each control. Further normalization by subtracting with minimum signal and dividing the resulting values with the maximum value within the dataset was applied to the melting curves that have clearly reached the steady state (i.e. no further change in signal at varying temperature), which could be due to experimental errors (e.g. plate reader, contaminated plate, pipetting error). The melting curve was fitted using explicit equation for bimolecular reaction of DNA hybridization (2) with assumptions that TAD roughly follows two-state transition, and concentrations of AC strands in large excess to S will not change much during the reaction. The fitted melting curve was used to obtain the first derivative curve as well as T_d

and ΔT (SI Table 2). For the kinetic experiment, the curve was fitted using exponential equation with an assumption that it follows pseudo-first order kinetic.

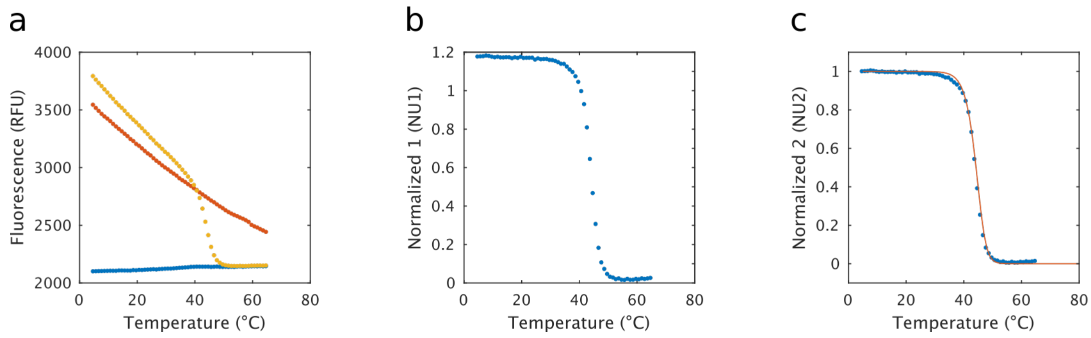

Figure S1. Normalization process of fluorescence signal for quantification of TAD activity. (a) Raw data from TAD characterization experiment (blue = negative control, red = positive control, yellow = experimental sample). (b) Background subtraction and normalization using negative and positive controls. (c) Further background subtraction and normalization using minimum and maximum value within the dataset (blue dot) followed by melting curve fitting (red line).

SI Table 2. T_d and ΔT of different AC designs obtained by fitting melting curves

Design parameters						***TAD properties***	
**#**	***N***	***L***	***C (μM)***	H_L	H_l	T_d	**ΔT**
1	0	n/a	10	4	4	58.5	27.4
1	0	n/a	10	4	10	50.3	25.7
1	0	n/a	10	6	4	62.0	31.2
1	0	n/a	10	8	4	60.9	35.1
1	0	n/a	10	10	4	61.3	40.2
1	2	6	10	6	4	43.7	21.7
1	2	8	10	6	4	49.0	21.6
1	2	10	3.16	6	4	48.1	19.4
1	2	10	10	6	4	51.5	18.9
1	2	10	31.6	6	4	53.9	17.5
1	4	8	10	n/a	n/a	44.3	16.6
1	6	10	10	6	4	32.2	29.1
2	2	6	10	6	4	23.8	19.9
2	2	8	10	6	4	37.8	18.2
2	2	10	10	6	4	46.6	15.8
2	4	8	10	n/a	n/a	27.2	19.2
2	4	10	10	n/a	n/a	39.9	15.2

SI2. Rationale for design utilizing association

As demonstrated by Rogers and Manoharan (3), simple designs without association can achieve reversible DNA strand displacement (DSD) by changing temperature. However, such designs lack flexibility of design parameters afforded by association in temperature-dependent associative DSD (TAD). To allow incumbent strand (I) to be completely single-stranded for downstream reactions, one possible design is a two-strand system with an initiator strand that binds toehold region of substrate strand (S), branch migrates, and displaces I via toehold exchange and an anchor strand that binds the rest of S and prevents reverse DSD of initiator strand by I at temperature below displacing temperature (T_d) (Figure S2a). Such design has to make sure that the duplex between initiator strand and I is thermodynamically unstable (i.e. shorter) compared to the duplex between I and S so that I-S pair dominates at temperature above T_d. This constraint consequently slows down the toehold exchange process since it requires either a short toehold for initiator strand or a long anchor strand. One such system was tested and showed temperature-dependent DSD behavior, but the kinetic was very slow, and it never reached 100% displacement at temperature below T_d with the protocol used in this experiment (Figure S2b). It is possible that the maximum displacement is around 90% since the concentration of anchor strand was 10 times the concentration of I. Moreover, the design parameters are not as flexible and straightforward as TAD to change the temperature-dependent behavior. Therefore, we used the TAD design that utilizes association.

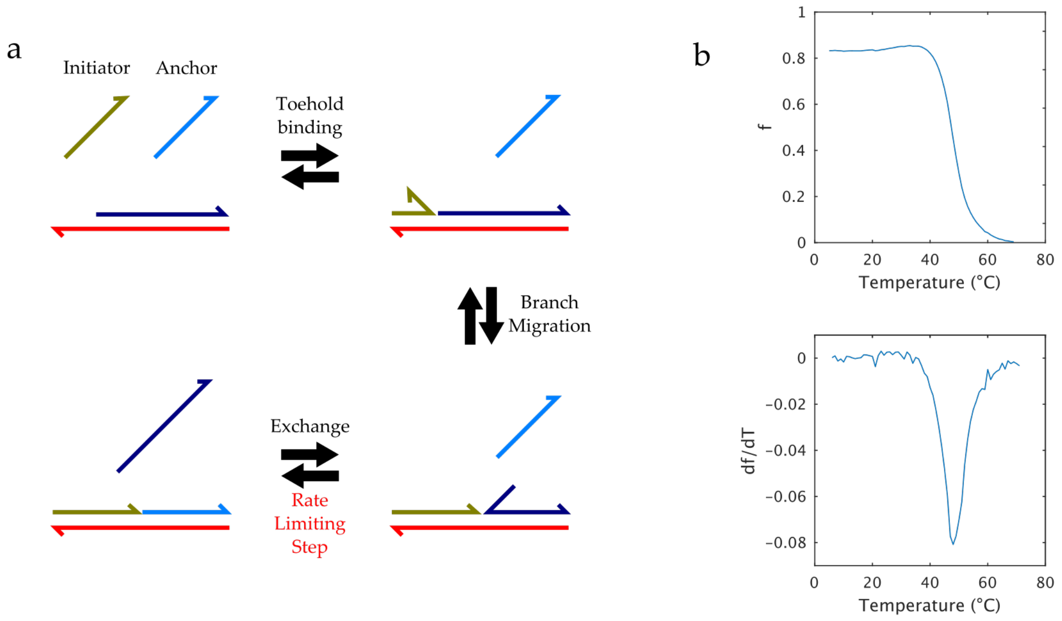

Figure S2. Design and characterization of two-strand system without association. (a) A schematic of the two-strand system and its possible reaction pathway. (b) Background subtracted and normalized melting curve of displacement fraction, f, and its first derivative with respect to temperature, df/dT, of the two-strand system.

SI3. Further discussion on design of TAD

The backward reactions (> T_d) are strand displacement in cases where the melting temperature of the central AC strand with S (i.e. β domain in Figure 1a) is higher than T_d. Therefore, TAD requires less stringent sequence design criteria than hybridization-based reaction unless the design is intentionally made otherwise (i.e. short or no β domain). Furthermore, hybridization crosstalk becomes less of an issue at higher temperatures. Although it has not been extensively explored in this work, the toehold exchange reactions using TAD are hindered due to the penalty of four-way junctions if the invading and dissociation toehold have the same free energy. However, the toehold strength can be tuned easily by changing sequence and the number of base pairs. With better understanding of energetic penalty of DNA four-way junctions, it should be possible to fine-tune the free energy of toehold for toehold exchange reactions at the specific temperature. The TAD designs tested in this manuscript are, in effect, performing toehold exchange at T_d (i.e. 50% displacement).

SI4. Discussion on choice of designs shown in Figure 2 of main text

In Figure 2a, we chose to show the data from I_2-S_2 instead of I_1-S_1 because they resulted in larger variations in T_d and ΔT (SI Table 2). We believe this drives home the point better. Regardless, the general trend remained the same for both experiments where longer stem lengths exhibited increased T_d and decreased ΔT. In Figure 2b, we showed the data from I_1-S_1 because we did not test a design with N = 0 with I_2-S_2. Moreover, the temperature-dependence of DNA four-way junction may have contributed to ΔT in addition to N (see SI5 and Figure S3), thus we believe that the data from I_1-S_1 represent the effect of N better. In Figure 2c, we chose the AC design with N = 4, L = 8, H_L = 6, and H_I = 4 for I_1-S_1 for convenience.

SI5. Further discussion on design parameters and their effects on TAD activity

Holding number of strands constant, a larger net number of base pairs increases both enthalpy and free-energy release of exchanging I-S with AC-S, resulting in narrower ΔT and higher T_d. Conversely, when in our designs we increase the number of net strands, we are motivated to do so in order to increase the number of net base pairs. The increase in number of strands by itself contributes to an unfavorable entropy loss, and therefore contributes to a lowering of T_d. An increase in the concentration of AC strands decreases this entropy loss; ΔT is mostly unaffected while T_d increases. However, the assumption that enthalpy change and entropy change is largely unaffected by temperature change does not seem to be true for DNA four-way junctions that we used in the design of TAD. Therefore, given a large enough change in T_d due to different strand concentrations, we expect that ΔT may be affected as well.

In our qualitative TAD model, we assumed temperature-independent enthalpy and entropy changes of DNA complex formation via assembly of AC strands on S that displaces I. This assumption was based on the fact that these thermodynamic parameters can be regarded as largely independent of temperature for regular DNA duplex formation (3). Though detailed thermodynamic properties of DNA junctions are not fully elucidated, decrease in temperature results in an unfavorable enthalpy change opposed by a favorable entropic change in DNA four-way junction compared to regular DNA duplex (4). We use DNA junctions to connect different TAD domains (complementary to α, β, and γ domains depicted in Figure 1a) in our designs, and therefore the decrease in enthalpy change is associated with lower T_d. As a consequence, an AC design with larger N (N = 4, L = 8, C = 10 μM) resulted in larger ΔT compared to an AC design with smaller N (N = 2, L = 8, C = 10 μM) using I_2-S_2, which operated at lower temperature compared to I_1-S_1 because of higher concentration of I_2 at 10 μM compared to I_1 at 1 μM (Figure S3). It should be also noted that there was a larger difference in T_d compared to I_1-S_1 (SI Table 2), which would result in comparatively unfavorable enthalpy change of the four-way junction that contributes to the sharpness of transition measured by ΔT.

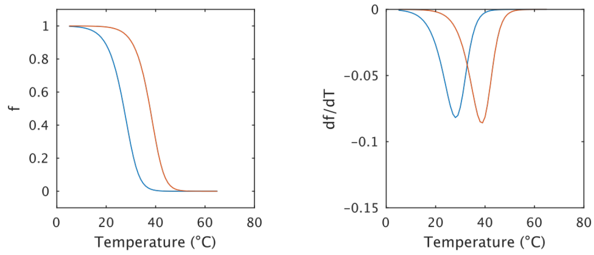

Figure S3. Effect of temperature-dependent thermodynamic parameters of four-way junction on TAD. Fitted melting curve (left) and its first derivative (right) of AC designs. An AC design with N = 4 (blue) has larger ΔT compared to an AC design with N = 2 (red), presumably due to decrease in enthalpy change that determines steepness of transition at lower T_d. f = fraction displaced.

In addition, the number of junction branches (M) is an associated parameter of N. In the current AC design, the number of DNA junctions is fixed at two, one between α and β domains and another between β and γ domains (Figure 1a). In order to increase N, M must increase to accommodate the assembly of more strands. For instance, M is 4 for four-way junctions used in all AC designs in the main text. However, M cannot be 4 for AC design with N = 6 because 5-strand system is maximum for AC design that has two junction points (Figure S4a). To make sure that all branches pair up to form base stacking, M must be even. Therefore, M has to increase to 6 in order to increase N to 6 in our current design. We have characterized 7-strand system (N = 7, L = 10, C = 10 μM, M = 6) using I_1-S_1 (Figure S4b). Lower T_d was observed compared to the system with the same design and experimental parameters except for lower N at 2. This result was as expected due to increased concentration-adjusted entropic penalty. However, the design resulted in much wider ΔT. In addition to the fact that enthalpy change of four-way junction is strongly dependent on temperature, it is possible that DNA junctions with more branches result in even stronger dependence of enthalpy change on temperature. Further study is necessary to determine whether or not M affects the behavior of TAD.

Though our first-generation TAD has demonstrated feasibility of utilizing heat energy as universal fuel for operation of DNA devices and circuit, it will be necessary to improve our ability to

quantitatively predict the behavior of TAD with given design and experimental parameters. Moreover, it will be necessary to achieve sharper ΔT and faster kinetics at low temperature (< 35°C) to imbue more complex behavior and being useful for applications that require faster response time at lower temperature. As demonstrated in this section, it is pertinent that we first understand the thermodynamic parameters of DNA junctions better, and the knowledge gained will lead to new designs with improved characteristics.

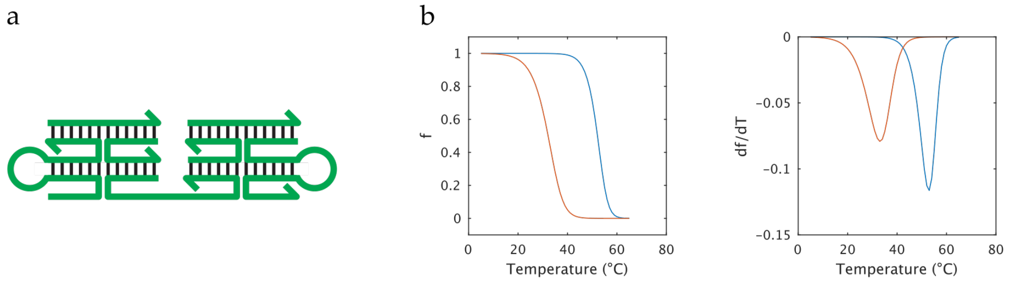

Figure S4. Characterization and comparison of 7-strand AC with 3-strand AC. (a) A schematic of AC design with $N = 6$. (b) Fitted melting curve (left) and its first derivative (right) of TAD activity. An AC design with $N = 2$ (blue) has smaller ΔT compared to an AC design with $N = 6$ (red). f = fraction displaced.

SI6. Effect of hairpin structure on AC design with *N* = 0

Our initial prediction was that the AC design with *N* = 0 would outcompete I at all temperature range since the melting temperature of the hairpin structures (H_L = 6, H_l = 4) were higher than I-S pair at 1 μM each. Surprisingly, the AC design was outcompeted by I with observed T_d at 62°C. Though predicted to be stable using Unafold (5), we hypothesized that the hairpin structures were actually unstable at higher temperature. To test this hypothesis, we analyzed the AC designs with varying length of hairpin stem (4, 8, and 10 bp) and the same length of hairpin loop (4 nt). If the hairpin structures were indeed unstable, the longer stem length should be able to shift the T_d to higher temperature. However, T_d remained constant even with longer hairpin stem, and T_d decreased with shorter hairpin stem (Figure S5a). Therefore, the transition at the hairpin stem length of 6 bp and above is most likely caused by a different reason, which could be the instability of four-way junction at this temperature range. In addition, we tested whether or not the longer hairpin loop could decrease T_d since longer hairpin loop should destabilize the hairpin structure, acting much like the concentration of each AC strand (*C*) for multi-strand systems. Indeed, the longer hairpin loop (10 nt compared to 4 nt) resulted in decreased T_d when the hairpin stem remained constant at 4 bp (Figure S5b). Therefore, the AC design with *N* = 0 can potentially be fine-tuned by changing the size of hairpin stem and loop.

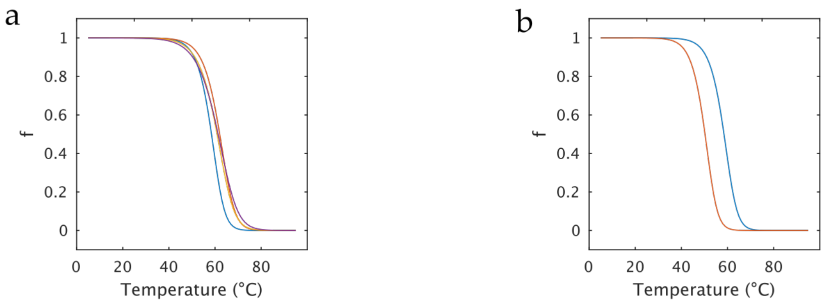

Figure S5. Characterization of design parameters of AC with *N* = 0. (a) Fitted melting curve of AC designs with varying hairpin stem length. An AC design with stem H_L = 4 (blue) is the only design with smaller T_d compared to AC designs with H_L = 6, 8, and 10 (red, yellow, and purple, respectively), which all have the same T_d though ΔT varied a little. (b) Fitted melting curve of AC designs with varying hairpin loop length. An AC design with H_l = 4 (blue) has higher T_d compared to a design with H_l = 10 (red). f = fraction displaced

SI7. Discussion on design of TAD circuit

The design of TAD circuit was chosen so that input signal can have greater freedom in terms of sequence constraint. The AC design with N = 2 used in previous experiments had hairpin structures in terminal AC strands where input strand would be placed. If the circuit were to be used to assess arbitrary nucleic acid input (e.g. mRNA), such constraint would be deleterious. Therefore, one of the hairpin structure was moved to the central AC strand. The basic architecture remains the same where two four-way junctions placed between toehold domains and central domain. In addition, we added “clamps” to eliminate one type of leaky reaction (6).

SI8. Discussion on kinetics of TAD

In case of toehold-mediated DSD, initial complex formed upon binding of toehold domain is transient, but the toehold binding initiates the subsequent branch migration and ends with displacement. Because of the fast kinetics of branch migration, the rate-limiting step for toehold-mediated DSD is the formation of toehold binding complex. In case of TAD, it can be viewed as multiple toehold binding events that leads to branch migration and displacement instead of a single binding event in toehold-mediated DSD. Moreover, the presence of DNA four-way junctions may affect the kinetics of branch migration at the junction point due to the energetic penalty of forming the junction. Therefore, TAD has slower kinetics compared to toehold-mediated DSD, but there still exists "fast" kinetic pathway compared to toehold-less exchange.

SI9. Supplemental References

1. Zadeh,J.N., Steenberg,C.D., Bois,J.S., Wolfe,B.R., Pierce,M.B., Khan,A.R., Dirks,R.M. and Pierce,N.A. (2011) NUPACK: Analysis and design of nucleic acid systems. *J. Comput. Chem.*, **32**, 170–173.
2. Böttcher,A., Kowerko,D. and Sigel,R.K.O. (2015) Explicit analytic equations for multimolecular thermal melting curves. *Biophys. Chem.*, **202**, 32–39.
3. Rogers,W.B. and Manoharan,V.N. (2015) Programming colloidal phase transitions with DNA strand displacement. *Science (80-.).*, **347**, 639–642.
4. Lu,M., Guo,Q., Marky,L.A., Seeman,N.C. and Kallenbach,N.R. (1992) Thermodynamics of DNA branching. *J. Mol. Biol.*, **223**, 781–789.
5. Markham,N.R. and Zuker,M. (2008) UNAFold: software for nucleic acid folding and hybridization. In *Methods in molecular biology (Clifton, N.J.)*.Vol. 453, pp. 3–31.
6. Qian,L. and Winfree,E. (2011) Scaling Up Digital Circuit Computation with DNA Strand Displacement Cascades. *Science (80-.).*, **332**, 1196–1201.
